# Supplementary material for: A prevalence-based transmission model for the study of the epidemiology and control of soil-transmitted helminthiasis
Source: PLoS One. 2022 Aug 25;17(8):e0272600. doi: 10.1371/journal.pone.0272600 (PMC9409602; doi:10.1371/journal.pone.0272600)
Supplement: S1 Appendix — (PDF) [file pone.0272600.s001.pdf]

Appendix: Bifurcation point of model (5)

Finding the bifurcation point, y_{bp}, of model (5) is equivalent to finding the critical point of $\mathcal{F}(y;k,z)$. Thus, we need to solve $d\mathcal{F}(y;k,z)/dy = 0$.

Denote $W_1 = (1-y)^{-\frac{1}{k}} - 1$. Then

$$\frac{2-z}{2}\left(1+\frac{2-z}{2}W_1\right)^{-(k+2)} = (1-z)\Big[1+(1-z)W_1\Big]^{-(k+2)},$$

$$\frac{2-z}{2}\left\{\left[\frac{2-z}{2(1-z)}\right]^{-\left(\frac{k+1}{k+2}\right)} - 1\right\}W_1 = 1 - \left[\frac{2-z}{2(1-z)}\right]^{\frac{1}{k+2}},$$

$$(1-y)^{-\frac{1}{k}} = \frac{\frac{z}{2-z}\left\{1-2\left[\frac{2-z}{2(1-z)}\right]^{\frac{1}{k+2}}\right\}}{\left[\frac{2-z}{2(1-z)}\right]^{-\left(\frac{k+1}{k+2}\right)} - 1},$$

$$y = 1 - \left(\frac{\left[\frac{2-z}{2(1-z)}\right]^{-\left(\frac{k+1}{k+2}\right)} - 1}{\frac{z}{2-z}\left\{1-2\left[\frac{2-z}{2(1-z)}\right]^{\frac{1}{k+2}}\right\}}\right)^{k}.$$

Thus, the bifurcation point of model (5) is defined as

$$y_{\text{bp}} = 1 - \left(\frac{\left[\frac{2-z}{2(1-z)}\right]^{-\left(\frac{k+1}{k+2}\right)} - 1}{\frac{z}{2-z}\left\{1-2\left[\frac{2-z}{2(1-z)}\right]^{\frac{1}{k+2}}\right\}}\right)^{k}.$$

By the Second Derivative Test, we find that

$$\begin{aligned}\frac{d^2\mathcal{F}(y;k,z)}{dy^2} =& \frac{2-z}{2}\left(\frac{k+1}{k}\right)^2\Big(1-y\Big)^{-\left(\frac{2k+1}{k}\right)}\left(1+\frac{2-z}{2}W_1\right)^{-(k+2)} \\ &- \Big(1-z\Big)\left(\frac{k+1}{k}\right)^2\Big(1-y\Big)^{-\left(\frac{2k+1}{k}\right)}\Big[1+(1-z)W_1\Big]^{-(k+2)} \\ &+ \frac{(k+1)(k+2)(1-z)^2}{k^2}\Big(1-y\Big)^{-2\left(\frac{k+1}{k}\right)}\Big[1+(1-z)W_1\Big]^{-(k+3)} \\ &- \frac{(k+1)(k+2)(2-z)^2}{4k^2}\Big(1-y\Big)^{-2\left(\frac{k+1}{k}\right)}\left[1+\frac{2-z}{2}W_1\right]^{-(k+3)}\end{aligned}$$

and

$$\begin{aligned}\left.\frac{d^2\mathcal{F}(y;k,z)}{dy^2}\right|_{y=y_{\text{bp}}} &= \frac{(k+1)(k+2)}{k^2}\Big(1-y\Big)^{-2\left(\frac{k+1}{k}\right)}\left(1+\frac{2-z}{2}W_1\right)^{-(k+3)} \\ &\quad\times\left[\Big(1-z\Big)^{\frac{k+1}{k+2}}\left(\frac{2}{2-z}\right)^{-\frac{k+3}{k+2}} - \left(\frac{2-z}{4}\right)^2\right] \\ &< 0,\end{aligned}$$

where the last inequality holds because the combination of terms in the square bracket is always negative for arbitrary k and z values. We therefore find that the maximum value of $\mathcal{F}(y;k,z)$ occurs at $y = y_{\text{bp}}$.
