# Supplementary material for: A prevalence-based transmission model for the study of the epidemiology and control of soil-transmitted helminthiasis
Source: PLoS One. 2022 Aug 25;17(8):e0272600. doi: 10.1371/journal.pone.0272600 (PMC9409602; doi:10.1371/journal.pone.0272600)
Supplement: Attachment — Submitted filename: ResponsesToReviewersComments.pdf [file pone.0272600.s002.pdf]

A prevalence-based transmission model for the study of the epidemiology and control of soil-transmitted helminthiasis

Dear Dr. Rodriguez,

Thank you for the reviews of our manuscript, which were extremely helpful. We have reworked and rewritten the manuscript in light of the comments. We have done everything the reviewers requested. Here is a point-by-point response to the reviewers.

Reviewer # 1

This reviewer noted that the manuscript is valuable and deserves to be published after some minor textual improvements. This reviewer had 5 minor comments.

Response: We have done everything this reviewer requested. Changes due to this reviewer are in red.

Comment (1): The purpose of the study is not in the abstract. Usually, standard formulations of STH transmission are not defined in terms of prevalence, but some of the authors have published papers on that. In fact, a previous work [23] on this subject is the starting point of the paper. This way, it is not clear in the abstract what are the novelties of the present work.

Response: Thank you very much for this observation. We have reworked the abstract to add the novelties of this work. (Page 1)

Comment (2): The same previous remark is also valid for some parts of the main text. The authors claim (lines 78-80) that their objective is to provide some analytical insights into the transmission dynamics of STH. This in fact is achieved throughout the paper, but the general idea of these insights is not provided in the introduction. What are the new and unique aspects of the work?

Response: We have added details to the introduction. (Page 3)

Comment (3): Could the authors be more precise and clear in the last sentence of the abstract? What do you mean by "complicated"? Please try to refer to the word "distribution" and explain it in more general terms for those who do not come from the field of applied mathematics. "It follows that control of soil-transmitted helminths will be **complicated** by the heterogeneity of worm **distribution**."

Response: This is an excellent suggestion. We have reworded the final sentence of the abstract. (Page 1)

Comment (4): The authors argue (lines 288-290) that their theoretical work has many applications. However, the precise meaning of applications is lacking here.

Response: You make a good point. We have added details of a project in Kenya to which this relates. (Page 30)

Comment (5): After Equation 3, please provide a reference for the described R0 (reference [23], for example).

Response: Done. (Page 5)

Reviewer # 2

This reviewer noted that the manuscript provides realistic insights into the soil-transmitted helminthiasis and that it is well-motivated and that both the model and the results are equally interesting. This reviewer request no changes.

In summary, we feel that these revisions have addressed all the points raised by the reviewers and hope that the manuscript is now acceptable.

Yours sincerely,

Nyuk Sian Chong, Robert Hardwick, Stacey Smith?, James Truscott and Roy Anderson
